# The significance of the subplate for evolution and developmental plasticity of the human brain

**DOI:** 10.3389/fnhum.2013.00423

**Published:** 2013-08-02

**Authors:** Miloš Judaš, Goran Sedmak, Ivica Kostović

**Affiliations:** Section of Developmental Neuroscience, Department of Neuroscience, Croatian Institute for Brain Research, University of Zagreb School of MedicineZagreb, Croatia

**Keywords:** cerebral cortex, neuron number, life-history, metabolic cost, subplate zone

## Abstract

The human life-history is characterized by long development and introduction of new developmental stages, such as childhood and adolescence. The developing brain had important role in these life-history changes because it is expensive tissue which uses up to 80% of resting metabolic rate (RMR) in the newborn and continues to use almost 50% of it during the first 5 postnatal years. Our hominid ancestors managed to lift-up metabolic constraints to increase in brain size by several interrelated ecological, behavioral and social adaptations, such as dietary change, invention of cooking, creation of family-bonded reproductive units, and life-history changes. This opened new vistas for the developing brain, because it became possible to metabolically support transient patterns of brain organization as well as developmental brain plasticity for much longer period and with much greater number of neurons and connectivity combinations in comparison to apes. This included the shaping of cortical connections through the interaction with infant's social environment, which probably enhanced typically human evolution of language, cognition and self-awareness. In this review, we propose that the transient subplate zone and its postnatal remnant (interstitial neurons of the gyral white matter) probably served as the main playground for evolution of these developmental shifts, and describe various features that makes human subplate uniquely positioned to have such a role in comparison with other primates.

## Introduction

We humans have large brains, and flatter ourselves to be smart. Accordingly, we are prone to think that “bigger is better” and to assume that larger (i.e., more encephalized) brains should have larger computational and cognitive abilities (for a comprehensive and historical review, see Herculano-Houzel, 2009, 2011a,b, 2012a). Some recent versions of this notion assume that improved cognition does not depend on relative brain size (i.e., the level of encephalization), but simply correlates with absolute brain size (Deaner et al., 2007) or with absolute numbers of cortical neurons and their connections and synapses (Roth and Dicke, 2005). However, if advantages of higher encephalization or increased brain size are so obvious, why big brains are so rare (Parker, 1990)? To answer this paradox, one should obtain more detailed knowledge on neural scaling rules in various mammalian orders (Herculano-Houzel, 2011a,b), as well as ask what the costs of encephalization are and how they can be afforded (Foley and Lee, 1991).

### Total number of neurons is more important than brain size *per se*

In a series of recent studies, starting with invention of new quantitative method for comparative analysis of cell and neuron numbers (Herculano-Houzel and Lent, 2005), it was clearly revealed that neural scaling rules evolved differently in different mammalian orders, such as rodents and lagomorphs (Herculano-Houzel et al., 2006, 2011; Herculano-Houzel, 2007), insectivores (Sarko et al., 2009), and primates (Herculano-Houzel et al., 2007; Gabi et al., 2010) including great apes (Herculano-Houzel and Kaas, 2011) and humans (Azevedo et al., 2009; Herculano-Houzel, 2009). These studies pointed out that, in terms of neuronal numbers, the human brain is linearly scaled-up primate brain and that our superior cognitive abilities might simply reflect the largest total number of neurons in the human brain (Herculano-Houzel, 2009, 2012a,b). It seems that basic primate advantage consists in packing more neurons in the same brain volume, thus avoiding prohibitively large increase in brain size (Herculano-Houzel, 2012a,b). In addition, human brains have ~3 times more brain neurons than gorillas and orangutans (Herculano-Houzel and Kaas, 2011). Another important finding concerns the coordinate increase in numbers of neurons in the cerebral cortex and cerebellum and the fact that the vast majority of all brain neurons are found in these two structures (Herculano-Houzel, 2009, 2010, 2011b, 2012a), thus supporting previous findings on cerebral-cerebellar co-evolution (Whiting and Barton, 2003; Ramnani, 2006; Ramnani et al., 2006; Balsters et al., 2010). Therefore, it has been proposed that “the larger the number of neurons in excess of that required to operate the body, the more complex and flexible the behavior of an animal can be expected to be, and thus the larger its cognitive abilities” (Herculano-Houzel, 2012a, p. 336).

### The brain is expensive tissue, and only mothers and infants of a “chosen primate” can afford to grow it beyond ordinary expectations

How much of the resting metabolic rate (RMR) is spend to maintain the adult brain? While most mammals expend 3–4% of RMR on brain metabolism (Mink et al., 1981; Armstrong, 1983, 1990), anthropoid primates spend about 8% of RMR to maintain their brains (Armstrong, 1983, 1990; Hofman, 1983a,b; Martin, 1983; Leonard and Robertson, 1992; Genoud, 2002). While the large brain-body-mass ratio of humans (the adult human brain is 2% of the body's mass) is not associated with elevations in RMR (Leonard and Robertson, 1992, 1994), adult humans nevertheless expend two to three times more energy on brain metabolism than other primates, that is 20–25% of RMR (Passmore and Durnin, 1955; Kety, 1957; Holliday, 1986; Aiello and Wheeler, 1995; Leonard et al., 1996; Rolfe and Brown, 1997; Genoud, 2002). These human brain costs are even more impressive during childhood, because the brain consumes roughly 87% of RMR in the newborn, and 44% in a 5 year old child (Holliday, 1986). In comparison to the neonate chimpanzee, the cost of the human neonate brain is significantly greater, and by the age of 5 years these costs are 3 times as great (Foley and Lee, 1991). Such energetic costs seem also to exert a selective pressure toward metabolically efficient neural morphology, that is, metabolically efficient patterning of dendritic arborizations (Wen and Chklovskii, 2008), neural codes (Levy and Baxter, 1996; Balasubramanian et al., 2001), and brain wiring patterns (Chen et al., 2006).

Positive pleiotropic gene effects on relative brain and body growth occur during prenatal and early postnatal periods, because genes affecting both traits generally do so during fetal and early postnatal growth, when both brain and body size are growing rapidly (Riska and Atchley, 1985).The fetal brain at any stage of development constitutes a markedly larger proportion of total fetal weight in primates than in other mammals (Sacher, 1982), and this difference is still observable in neonates (Martin, 1983). However, this difference is no longer clear in comparisons among adults, due to differential postnatal changes in different mammals (Martin, 1983). This points to the crucial importance of brain development (Martin, 1996). For example, evolutionary shifts in brain development lead to differences in development of social behavior and cognition even between such closely related species such as chimpanzees and bonobos (Wobber et al., 2010).

The growth of the brain significantly depends upon energetic and hence ecological conditions (Martin, 1983; Foley and Lee, 1991); as succintly stated by Foley and Lee (1991, p. 223): “whatever selective pressures there may be driving the size of the brain up, these are satisfied only in the context of there being sufficient energy.” Having a large brain imposes additional energetic costs on both the infant and the mother; the mother can derive that energy either from the incorporation of higher quality food, from feeding for longer each day, or from maintaining lactation over a longer period (Foley and Lee, 1991). Thus, the evolution of a large brain requires that energetic constraints are lifted (Armstrong, 1983; Hofman, 1983a,b, 1993; Martin, 1983, 1996; Foley and Lee, 1991; Leonard and Robertson, 1992, 1994; Aiello and Wheeler, 1995; Leonard et al., 2003; Isler and van Schaik, 2006a,b, 2009).

Some recent evidence suggests that the metabolic cost may be an even more limiting factor to brain expansion than previously suspected (Herculano-Houzel, 2012b). Namely, the estimated glucose use per neuron is remarkably constant, varying only by 40% across the six species of rodents and primates, including humans (Herculano-Houzel, 2011c). Thus, it seems that the brain energy budget per neuron is fixed across species and brain sizes and that the total metabolic cost of a brain is a simple, direct function of its number of neurons (Herculano-Houzel, 2011c). These findings clearly suggest that neuronal metabolism imposes a series of constraints upon brain structure, function, and evolution (Herculano-Houzel, 2011c, 2012b; Fonseca-Azevedo and Herculano-Houzel, 2012). The metabolic constraints upon brain scaling in evolution are imposed by absolute number of neurons, because adding neurons to the brain comes at a sizable cost of 6 kcal/d per billion neurons (Herculano-Houzel, 2011c).

Three major hypotheses have been proposed to explain how larger brains are afforded among mammalian species (see Jones and MacLarnon, 2004, for a comprehensive review): direct metabolic constraint hypothesis (Armstrong, 1983; Hofman, 1983a,b); the expensive tissue hypothesis (Aiello and Wheeler, 1995); and the maternal energy hypothesis (Martin, 1981, 1983, 1996, 2007; Martin et al., 2005). None of these hypotheses has the general applicability in multiple mammalian clades with different evolutionary histories (Jones and MacLarnon, 2004), and there are several strategies for meeting the energetic demands of encephalization which can be manifested differentially across taxa (Barrickman and Lin, 2010). However, at least in the case of large-brained apes and humans, the maternal energy hypothesis seems to be well supported by the available evidence (Martin, 1996; Isler and van Schaik, 2006a,b, 2009; Isler et al., 2008). This hypothesis proposes that the brain size is constrained by the amount of energy that a mother can provide during the early stages of her offspring's ontogeny (Martin, 1996, 2007). It should be noted that such a primary link between the mother's metabolic capacity and the developing brain of her offspring allows other variables to influence ultimate adult brain size (Martin, 1996). In addition, there may be no very tight relationship between relative brain size and specific behavioral capacities, and an increase in brain size may be advantageous in a diffuse fashion, i.e., may have some kind of permissive or promotive influence with respect to the evolution of cognition (Martin, 1996).

The encephalization is also associated with prolonged duration of most life-history stages, especially in primates (Sacher and Staffeldt, 1974; Harvey and Clutton-Brock, 1985; Barton, 1999; Kappeler and Pereira, 2003; Leigh, 2004; Barrickman et al., 2008) including humans (Bogin, 1997, 1999, 2009; Leigh, 2001). At least three changes in developmental timing occurred during the evolution of human encephalization: extended brain growth, retarded postnatal body growth, and a derived brain growth allometry (Vinicius, 2005). The human brain achieves its final size more by lengthening the time of growth than by adopting an unusual rate of growth (Passingham, 1985). The prolonged period of growth in humans may be partly an adaptation to limit the already high total and brain energy requirements during childhood (Leonard and Robertson, 1992). Others have suggested that selection has acted to decrease human somatic growth rates during childhood and juvenility (in comparison to chimpanzees), to help fuel the energy-expensive brain and to allow more time for increased cognitive development with lower body-maintenance costs (Walker et al., 2006).

So, how our evolving ancestors have solved the above mentioned energetic challenges? Obviously, there were a number of step-wise changes, stretching perhaps over last 2 million years, i.e., during the evolution of the genus *Homo*. One part of the solution seems to be a significant change in dietary and foraging habits, as humans have a much higher quality diet than expected for their size or their resting metabolic needs (Leonard and Robertson, 1994, 1997; Fish and Lockwood, 2003). Turning to animal source foods, such as meat, as a routine dietary component probably represented an important step (Milton, 1999, 2003). However, it seems that a diet relying solely on consumption of raw food was not sufficient to remove this metabolic constraint on the increase of brain size—as documented in a recent study, the largest great apes cannot afford both a large body and a larger number of brain neurons (Fonseca-Azevedo and Herculano-Houzel, 2012). The use of fire and the invention of cooking might have a substantial role, because the cooking increases enormously the energy yield of foods and the speed with which they are consumed (Carmody and Wrangham, 2009; Carmody et al., 2011). While raw meat increased the caloric content of the diet of early hominids (Milton, 1999), the cooked meat is easier to chew and has a higher caloric yield (Carmody et al., 2011). In fact, as the metabolic cost is limiting enough to impose tradeoffs in brain evolution (Fonseca-Azevedo and Herculano-Houzel, 2012), the invention of cooking food was probably necessary to overcome such a metabolic limitation in the human lineage (Wrangham et al., 1999; Wobber et al., 2008; Carmody and Wrangham, 2009; Carmody et al., 2011). It should be also noted that there is evidence of up-regulation of genes related to energy metabolism in human evolution (Grossman et al., 2001; Cáceres et al., 2003; Uddin et al., 2004).

Another part of the solution seems to be represented by profound changes in the human life-history (Bogin, 1997, 1999, 2001, 2009; Hawkes et al., 1998; Kaplan et al., 2000; Crews, 2003; Leigh, 2004; Gurven and Walker, 2006; Walker et al., 2006). There are several hypotheses on the evolution of human life-history, such as the grandmother hypothesis (Hawkes et al., 1998), the embodied capital hypothesis (Kaplan et al., 2000), the reserve capacity hypothesis (Crews, 2003; Larke and Crews, 2006), and the reproductive fitness hypothesis (Bogin, 1997, 1999, 2009). Briefly, primates and other social mammals have three postnatal life history stages: infancy, juvenile and adult (Pereira and Fairbanks, 1993). However, human life history is characterized by the addition of childhood, adolescence, and grandmotherhood (postmenopausal stage) as biologically, behaviorally, and mathematically definable stages of the life cycle (Bogin, 1997, 1999; Hawkes et al., 1998). The transition from infancy (birth to 30–36 months) to childhood is characterized by weaning and the completion of deciduous tooth eruption (Bogin, 1999, 2001). During the childhood, older members of the social group acquire, prepare, and provision foods to children, and this style of cooperative care represents a major evolutionary invention in the human life-history (Bogin, 1999, 2009). The adolescence includes the years of postpubertal growth (10–18 years for girls, 12–21 years for boys) (Bogin, 1999, 2001).

It is important to note that the childhood and adolescence stages of human life history evolved due to the selective advantages for increased fertility and reproductive fitness of mothers (Bogin, 1999, 2001, 2009), while the benefits of these stages for increased brain growth and learning are important, but secondary, outcomes (Bogin, 2009). In summary, the human species has more life stages than any other mammal and more time for growth and development than any primate (Bogin, 2009). Another important evolutionary novelty in human life-history is that human food provisioning and care to children and their mothers goes beyond the cooperative breeding of other mammals—humans use biological relationships and also marriage, systems of economic exchange, political power structure, and gender-role construction (Bogin, 2009). In other words, human life-history is culturally patterned (Bogin, 2001, 2009; Crews, 2003). Such investments of energy and care from prenatal to early adult life stages build a greater level of reserve capacity than found in any other primate (Crews, 2003; Larke and Crews, 2006; Bogin, 2009).

### The subplate is critically involved in the ontogenesis of the human cerebral cortex

The data reviewed above clearly suggest that the developing brain played significant role in the evolution of the human life-history. As the telencephalon and the cerebral cortex represent by far the largest part of the human brain, we here focus on the potential evolutionary role of the transient subplate zone, because it is critically involved in the development of the primate and human cerebral cortex (Bystron et al., 2008) and it reached a peak of its evolutionary prominence in the human brain (Kostovic and Rakic, 1990; Molnár et al., 2006; Rakic, 2006; Bystron et al., 2008). The role of the subplate in the development and plasticity of the cerebral cortex has been already well described in a number of excellent reviews (Allendoerfer and Shatz, 1994; Kostović and Judaš, 2002, 2006, 2007, 2010; Kanold and Shatz, 2006; Molnár et al., 2006; Rakic, 2006, 2009; Bystron et al., 2008; Kanold and Luhmann, 2010; Clowry et al., 2010; Judaš, 2011). Therefore, we will here only briefly review those aspects of the human subplate which are directly relevant for understanding of our present thesis. As the subplate development in the human brain has also been extensively illustrated in our previous publications (Kostovic and Rakic, 1980, 1990; Kostović and Judaš, 2002, 2006, 2007, 2010; Judaš, 2011), we here provide only a few figures aimed to enhance the understanding of our main argument.

**Figure 1:**
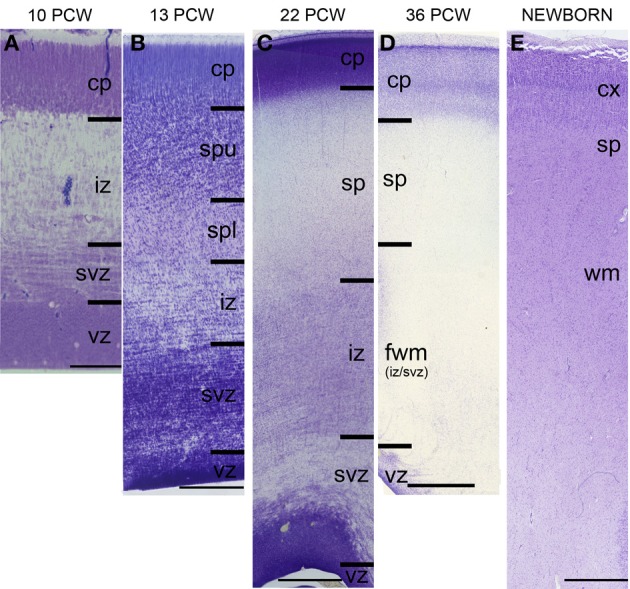
**Laminar development of human telencephalon from 10 postconceptional weeks (PCW) to newborn**. The layers are transient and their appearance changes with changes in neurogenetic events. The subplate starts to develop around 13 PCW, reaches the peak of its development between 22 and 24 PCW, and starts to resolve around 34 PCW. In the newborn brain, the subplate remains during the first year, when the subplate disappears as a zone but its neurons become incorporated into the subcortical white matter as so-called interstitial neurons. cp, cortical plate; sp, subplate zone; iz, intermediate zone; svz, subventricular zone; vz, ventricular zone. Bar = 100 μm **(A)**, 250 μm **(B)**, 1 mm **(C–E)**.

**Figure 2:**
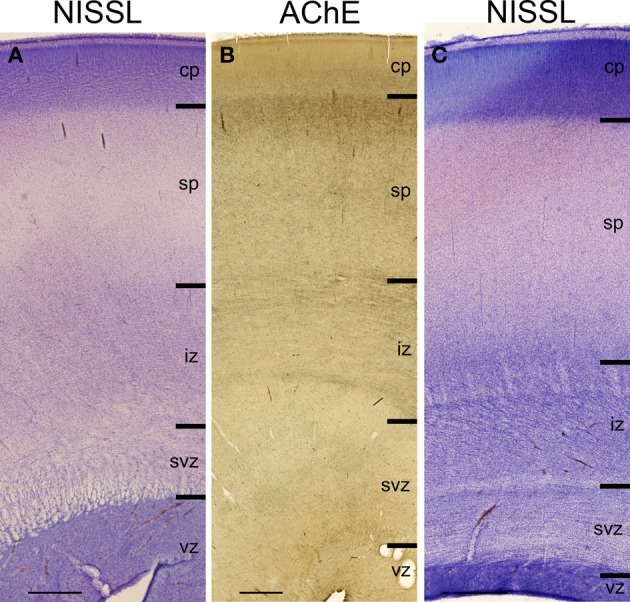
**Lamination of frontal (A), mid-central (B) and occipital (C) region of human telencephalon at the peak of subplate development (22–24 PCW), as revealed by Nissl staining and acetylcholinesterase (AChE) histochemistry**. At the peak of subplate development (22–24 PCW), subplate zone is the largest compartment of the human telencephalon. It is the place of intense synaptic activity and “waiting” compartment for the thalamocortical fibers (dark band below cp). Note that there are regional differences in the lamination between frontal and occipital region. cp, cortical plate; sp, subplate zone; iz, intermediate zone; svz, subventricular zone; vz, ventricular zone. Bar = 1 mm.

**Figure 3:**
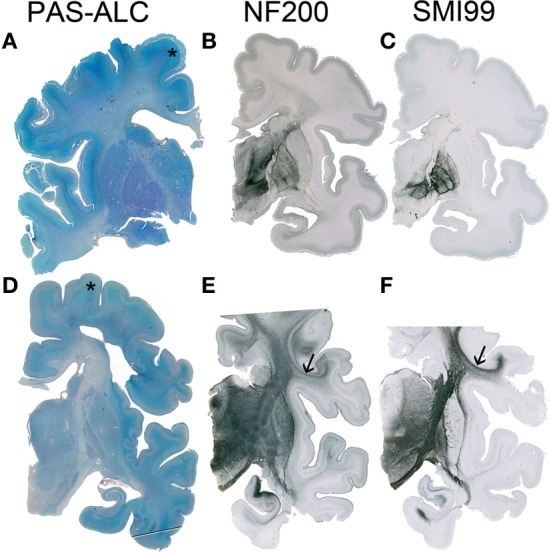
**Although the resolution of the subplate zone starts after 34 PCW, the subplate remains visible as a major component of the telecephalic wall (asterisk in A,D)**. In the human telencephalon, cortico-cortical connections are still not developed **(B)** or myelinated **(C)** at 33 PCW, while at 40 PCW substantial development and myelination of cortico-cortical fiber can be observed (arrow in **E,F**). **A–C** 34 PCW, **D–F** 40 PCW.

**Figure 4:**
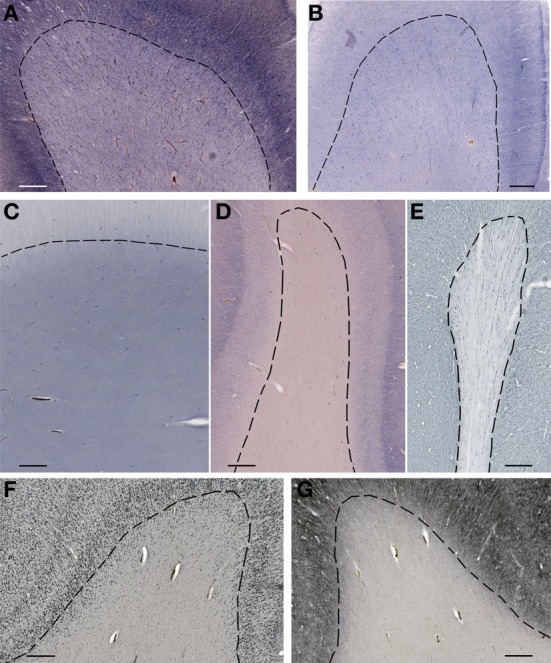
**Subplate and white matter interstitial neurons stained for NOS (NADPH-diaphorase-stained neurons in A–D), MAP2 (E,G) and NeuN (F) are visible throughout the subplate (A,B) and the white matter (C–G)**. Note that subplate/white matter interstitial neurons are numerous even after the first year of life, when subplate zone disappears. **(A)**, 37 PCW; **(B)**, 13 days; **(C)**, 12 years; **(D)**, 57 years; **(E,G)**, 13 months; **(F)**, 51 years. Bar = 1 mm.

**Figure 5:**
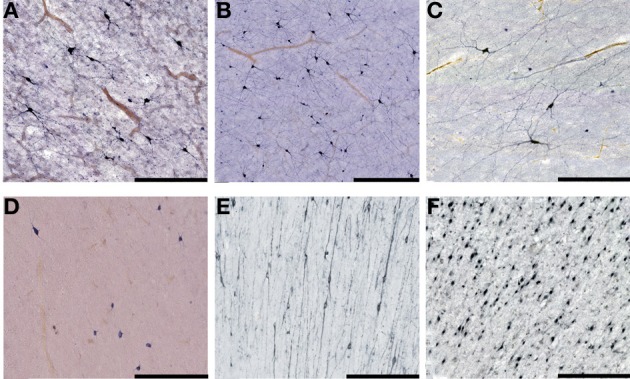
**Higher magnification view of subplate and white matter interstitial neurons displayed in panels A–F of the Figure 4 and stained for NOS (NADPH-diaphorase-stained neurons in A–D), MAP2 (E) and NeuN (F)**. Note that dendritic arborizations of subplate/interstitial neurons continue to grow and develop even after the disapearance of the subplate zone during the first year of life (compare (**A** and **B** with **C**). **(A)**, 37 PCW; **(B)**, 13 days; **(C)**, 12 years; **(D)**, 57 years; **(E)**, 13 months; **(F)**, 51 years. Bar = 0.5 mm.

The subplate zone was first described in the human fetal brain (Kostović and Molliver, 1974; Judaš et al., 2010a; see, for a comprehensive historical review). The subplate is the largest transient compartment of the fetal neocortical anlage (see Judaš, 2011, for a comprehensive review). The human subplate develops between 13 and 15 postconceptional weeks (PCW), remains the largest compartment of the neocortical anlage between 15 and 30 PCW, and begins slowly to disappear toward the end of gestation and during the early postnatal period (Figure 1). The developmental peak of the subplate is reached during midgestation, when it is about four times thicker than the cortical plate (Figure 2). It should be noted that the subplate is still present in the newborn brain during the period when various corticocortical connections continue to develop (Figure 3). Finally, many subplate neurons survive postnatally and eventually transform into interstitial neurons of the subcortical (gyral) white matter of the adolescent and adult brain (Figures 4, 5) (Kostovic and Rakic, 1980, 1990; Judaš et al., 2010b). While the dissolution of subplate begins during the last third of gestation, it remains present (as recognizable architectonic compartment) under the prefrontal and other association cortices up to 6 postnatal months (Kostovic and Rakic, 1990). It should be noted with a great regret that there are no data available on the subplate of great apes; in fact, there are no histological data on any aspect of prenatal cortical development in great apes.

The subplate contains numerous neurons of various morphological types (Mrzljak et al., 1988, 1990, 1992) and molecular phenotypes, including differentiated projection (glutamatergic) neurons and local (GABA and peptidergic) interneurons (Judaš et al., 1999, 2010b; Judaš, 2011). It also serves as a waiting compartment for growing cortical afferents (Rakic, 1977; Kostovic and Rakic, 1990). Various afferent fibers sequentially grow into the subplate, establish temporary synaptic circuits, and “wait” in the subplate for several months before relocating into their final target, the cortical plate (Kostović and Goldman-Rakic, 1983; Krmpotić-Nemanić et al., 1983; Kostovic and Rakic, 1984, 1990; Kostović, 1986). After 28 PCW, waiting associative and commissural pathways are major constituents of the subplate (Kostovic and Rakic, 1990; Kostović et al., 2008; Kostović and Judaš, 2009). While long corticocortical pathways begin to develop in the early fetal period (Vasung et al., 2010), the development of short corticocortical connections is very protracted and lasts for at least 1 year after birth (Kostović et al., 2012). It should be noted that cortical pyramidal neurons also require about 3 years of postnatal development in order to attain their adult-like size of dendritic arborization (Petanjek et al., 2008).

The subplate is also the major site of synaptogenesis in the midfetal brain (Molliver et al., 1973; Kostovic and Rakic, 1990) and contains diverse and transient neuronal circuits which represent a neurobiological basis for transient electrophysiological and behavioral phenomena in fetuses and early preterm infants (Kostović and Judaš, 2002, 2006, 2007, 2010). Although the onset of cortical synaptogenesis is an early fetal event (Molliver et al., 1973; Kostovic and Rakic, 1990), it should be noted that cortical synaptogenesis is predominantly postnatal process and that synaptic overproduction and developmental plasticity in the human cortex continue for at least 20 years (Petanjek et al., 2011).

The transformation of the fetal white matter occurs gradually and in parallel with gradual dissolution of the subplate, and continues postnatally (Judaš, 2011). The period spanning the last prenatal month and at least the first postnatal year is characterized by significant fiber-architectonic reorganization at the cortical/white matter interface (Kostović et al., 2012). This reorganization is related to the postnatal persistence of the subplate remnant, the onset of myelination, the appearance of tertiary gyri and sulci, development of short corticocortical connections (Kostović et al., 2012), and probably other factors, such as changes in microvascular network, changes in molecular profile of the extracellular matrix, development of white matter astrocytes, and so forth (Judaš, 2011).

Thus, histogenetic processes in the human fetal and perinatal brain are protracted and significantly overlap (Judaš, 2011), but the subplate represents a playground for the majority of important events during that developmental window. The functional significance of transient fetal circuitry and the pivotal role of the subplate have already been extensively reviewed in both experimental model animals (Allendoerfer and Shatz, 1994; Kanold and Luhmann, 2010) and in humans (Kostović and Judaš, 2006, 2007, 2010; Judaš, 2011). Therefore, it will suffice to point out that the human perinatal and early postnatal period is characterized by simultaneous existence of two separate (but interconnected) types of cortical circuitry organization: (a) transient fetal circuitry, centered at the subplate zone, and (b) immature but progressively developing permanent cortical circuitry, centered at the cortical plate (that is, developing cortical layers I-VI). Thus, the developing human cortex passes through three major early stages of functional development (Kostović and Judaš, 2006, 2007, 2010): (1) initial fetal circuitry which is endogeneously (spontaneously) driven, (2) perinatal dual circuitry (co-existence of endogeneously driven subplate-centered transient circuitry with developing cortical plate-centered permanent circuitry) and (3) postnatally established permanent (externally driven) cortical circuitry (Judaš, 2011).

### The subplate as the playground for evolution of cortical development

While the focus of this review is on putative (and relatively recent) evolutionary changes of the subplate in the primate and hominid lineage, it is important to note that the subplate may have a much older phylogenetic origin. As pointed out in several recent studies (Montiel et al., 2011; Wang et al., 2011), there are currently three hypotheses about the phylogenetic origin of subplate neurons: (1) subplate neurons were all already present in the common ancestor of mammals and sauropsids (e.g., Marin-Padilla, 1978; Aboitiz et al., 2005); (2) subplate may be unique to mammals and represent an embryonic adaptation to support development of increasingly complex neocortex (Kostovic and Rakic, 1990; Supér and Uylings, 2001; Molnár et al., 2006); and (3) the subplate in mammals may represent a combination of new and ancestral cell populations (Aboitiz, 1999; Aboitiz et al., 2005; Wang et al., 2011; Montiel et al., 2011). The third hypothesis suggests that, although embryonic subplate cells were present in the common ancestor of both mammals and sauropsids, additional populations of subplate cells evolved in mammals as the neocortex became progressively larger and more complex (Montiel et al., 2011; Wang et al., 2011). As the evolution of the mammalian cortex required the modification of developmental programs, it seems probable that some of these started to rely on novel populations of subplate neurons possibly characterized by different targets of connectivity (Kostovic and Rakic, 1990; Montiel et al., 2011). Thus, it is important to determine if and how the subplate has been altered in distinct mammalian lineages and to perform comparative gene expression profiling studies of subplate neurons in different species (Osheroff and Hatten, 2009; Wang et al., 2010, 2011; Oeschger et al., 2012; Hoerder-Suabedissen et al., 2013). For example, species-specific differences in subplate markers have been described even between rat and mouse (Wang et al., 2011). In addition, in primates, in contrast to rodents, neurons are continuously added to the subplate throughout cortical neurogenesis (Smart et al., 2002; Lukaszewicz et al., 2005; Molnár et al., 2006). Finally, in addition to the increased number of neurons in the human subplate (Kostovic and Rakic, 1990; Smart et al., 2002; Bystron et al., 2008), there is both an increased complexity of subplate cell types (Kostovic and Rakic, 1990; Mrzljak et al., 1988, 1990, 1992; Wang et al., 2010) and subplate arrangements including the superficial vs. deep compartmentalization of human subplate neurons (Wang et al., 2010).

Thus, the available evidence suggests that human subplate contains an increased number of (ancestral and derived) subplate neurons as well as increased diversity of a derived population of subplate neurons. As these neurons are active and therefore metabolically expensive, the potential increase in number of subplate neurons was probably subject to a significant selective pressure due to above described metabolic constraints.

The lift-up of metabolic constraints by hominid ancestors opened new vistas for the developing brain, because it became possible to metabolically support transient patterns of brain organization as well as developmental brain plasticity for much longer period and with much greater number of neurons and connectivity combinations in comparison to apes. We propose that the transient subplate zone and its postnatal remnant (interstitial neurons of the gyral white matter) probably served as the main playground for evolution of these developmental shifts, for the following reasons.

First, as described above, the human brain contains about three times more neurons than the brain of apes. As monkey and human cortical neurons are all generated before birth (Rakic, 2006, 2009; Bystron et al., 2008), and newborn human brain is also significantly larger than that of newborn apes (ca. 350 vs. ca. 200 g), it is logical to conclude that brains of human newborns also contain greatly increased number of neurons in comparison to newborn apes. By extension, even if we assume that apes have proportionately equally developed subplate, humans would still have more numerous subplate neurons. Moreover, that huge number of subplate neurons is actively involved in shaping of cortical circuitry for at least 12 months (Judaš, 2011; Kostović et al., 2012), and large number of subplate neurons survives into adolescence and adulthood as subcortical interstitial neurons (Judaš et al., 2010b). Thus, significantly enlarged number of key players in developmental cortical plasticity is present and metabolically supported to play this game for much longer than in any other primate species.

Second, as also described above, the subplate serves as a “waiting” compartment for numerous contingents of ingrowing cortical afferents. The human subplate contains the largest amount of both subcortical and corticocortical waiting afferents, during the longest developmental period. The subplate is the major site of synaptogenesis and early circuit formation during the prenatal period. Its circuitry also coexists with initial adult-like circuitry during the perinatal period, and its neurons continue to be involved in the development of short corticocortical connections during the first postnatal year (Kostović et al., 2012). Thus, humans become able to sustain an extremely long period of cortical circuitry development, characterized by large overproduction of axonal and dendritic branches, synapses and reorganizational events in response to environmental influences. This includes the shaping of cortical connections through the interaction with infant's social environment, which probably enhanced typically human evolution of language, cognition and self-awareness.

In summary, we propose that life-history changes that enabled the metabolic sustainability of prolonged retention of the subplate also provided the playground for prolonged and more diverse perinatal and early postnatal plastic interactions between the increased number of subcortical and corticocortical afferents and increased number of cortical neurons (including the perinatal co-existence of fetal and adult-like cortical circuitry). This enabled the evolution of new types of modular, areal and connectional organization of the human cerebral cortex, subserving cognition and language. Our proposal is also in agreement with the reserve capacity hypothesis (Crews, 2003; Larke and Crews, 2006) and the reproductive fitness hypothesis (Bogin, 1997, 1999, 2001, 2009), because the increased reserve capacity of human species (in comparison to apes) clearly enables the longer development of the human brain, with significant consequences for learning and socialization as well as plasticity and recovery after brain lesions.

### Conflict of interest statement

The authors declare that the research was conducted in the absence of any commercial or financial relationships that could be construed as a potential conflict of interest.
